# Ginkgolide B Alleviates Airway Inflammation in Hyperoxia Lung Injury

**DOI:** 10.1002/iid3.70364

**Published:** 2026-04-02

**Authors:** Xuesong Wang, Mingwu Chen, Yong Lv, Yue Qi, Dake Huang, ShuShu Wang

**Affiliations:** ^1^ Department of Pediatrics, The First Affiliated Hospital of USTC, Division of Life Sciences and Medicine University of Science and Technology of China Hefei Anhui China; ^2^ Department of Pathology Anhui Medical University Hefei Anhui China

**Keywords:** bronchopulmonary dysplasia, Ginkgolide B, inflammation, platelet activation factor

## Abstract

**Background:**

Bronchopulmonary dysplasia (BPD) is a chronic lung disease characterized by inflammation, often caused by prolonged hyperoxia exposure in preterm infants. Platelet‐activating factor (PAF) is a potent inflammatory mediator, but its role in BPD is unclear. This study explores the therapeutic potential of the PAF receptor antagonist Ginkgolide B (GB) in hyperoxia‐induced lung injury.

**Methods:**

We observed the activation status of platelets and neutrophils in BALF（bronchoalveolar lavage fluid) of patients with BPD. The supernatant of BALF from patients was sent for protein chip detection and validated by ELISA. We also measured the levels of PAF in peripheral blood and BALF. In vitro experiments, we stimulated neutrophils with PAF to detect the expression of inflammatory cytokine genes. Finally, we established a neonatal rat model of hyperoxia‐induced lung injury mimicking BPD and intervened with GB.

**Results:**

We observed early activation of platelets and neutrophils in the peripheral blood of BPD patients, with elevated plasma levels of PAF and myeloperoxidase‐DNA (MPO‐DNA). In BALF, neutrophils showed increased CD66b and MPO expression. In vitro, PAF stimulation decreased CD62L expression on neutrophils and upregulated IL‐6 mRNA. Elevated levels of PAF, IL‐6, IL‐8, and GM‐CSF were found in BPD infants' BALF supernatant. Using animal models of BPD, we found that GB significantly reduced lung damage in hyperoxia‐exposed rats. GB decreased neutrophil‐derived IL‐6 and downregulated key proteins in the IL‐6 signaling pathway (pJak2 and pStat3).

**Conclusion:**

Our study demonstrates that PAF activates neutrophils and promotes their lung residence. GB effectively inhibits neutrophil‐derived IL‐6 and alleviates hyperoxia‐induced lung damage. Targeting the PAF pathway with GB may be a promising strategy for BPD and other hyperoxia‐related lung diseases.

**Abbreviations**
BALF: bronchoalveolar lavage fluid
BPD: bronchopulmonary dysplasia
GB: Ginkgolide B
GM‐CSF: granulocyte‐macrophage colony‐stimulating factor
GRO: growth‐related oncogene
IL: interleukin
KC: CXCL1
LCN2: lipocalin 2
MMP12: matrix metallopeptidase 12
MPO: myeloperoxidase
NICHD: National Institute of Child Health and Human Development
NO: nitric oxide
PAF: platelet activation factor
PGE2: prostaglandin E2

## 1 Introduction

Bronchopulmonary dysplasia (BPD) is a severe respiratory complication in premature infants, significantly impacting lung function and quality of life into adulthood. Characterized by arrested lung growth, simplified alveoli, and impaired vascular development [1, 2, 3], BPD has a multifactorial etiology that includes genetic susceptibility, prenatal and postnatal infections, high‐concentration oxygen inhalation during the perinatal period, and invasive ventilator support. Ultimately, the inflammatory response is the core pathogenesis of BPD. Therefore, identifying effective and safe treatment strategies is of utmost urgency.

In recent years, platelet‐activating factor (PAF) has emerged as a potent phospholipid mediator with well‐documented pro‐inflammatory properties, particularly in activating platelets and leukocytes. PAF exerts a wide range of biological activities through its receptors in various physiological and pathological processes, including coronary artery disease, asthma, rheumatoid arthritis, and sepsis [4, 5, 6, 7, 8]. Ginkgolide B (GB), a potent PAF receptor antagonist derived from Ginkgo biloba [9, 10], has demonstrated significant anti‐inflammatory effects in both in vitro and in vivo studies [9, 11, 12, 13, 14, 15, 16, 17, 18, 19, 20, 21, 22]. Initially used in cardiovascular and cerebral thrombotic diseases, GB has demonstrated efficacy in reducing inflammation by inhibiting key signaling pathways such as TLR2/4 and PI3K/Akt. It has also shown beneficial effects in conditions like renal failure, bronchoconstriction after lung transplantation, and ischemia/reperfusion injury [22].

Additionally, GB has been reported to inhibit eosinophil/neutrophil infiltration, reduce IL‐5/IL‐13/MPO levels, and alleviate alveolar hemorrhage in OVA‐induced asthma [23] and LPS‐induced lung injury models [24]. These effects suggest it may protect the immature alveolar‐capillary barrier by suppressing inflammatory cell recruitment and cytokine release—a key pathological process in BPD.

However, the relationship between PAF and BPD, as well as the potential of GB to inhibit key inflammatory pathways in hyperoxia‐induced lung injury, remains largely unexplored. We aimed to investigate whether GB could reduce lung inflammation by limiting PAF‐mediated neutrophil infiltration.

In this study, we first investigated platelet activation in the peripheral blood of preterm infants with BPD during the first postnatal week, as well as neutrophil activation in bronchoalveolar lavage fluid (BALF) samples. We then identified the relation of PAF and neutrophils, followed by measuring the concentrations of PAF and other inflammatory cytokines in both plasma and BALF supernatants of preterm neonates. Furthermore, we evaluated the efficacy of a novel therapeutic strategy using a neonatal rat hyperoxia model mimicking BPD and demonstrated that GB can effectively alleviate hyperoxia‐induced lung injury and downregulate the IL‐6/STAT3 signaling. These results provide new insights and potential avenues for developing safer and more effective treatments for BPD.

## 2 Materials and Methods

### 2.1 Restropective Analysis

A retrospective analysis of the neutrophil and platelet counts in the peripheral blood of preterm neonates with BPD and those without BPD was conducted, including 100 cases of BPD and 126 cases of non‐BPD based on 2001 NICHD diagnostic criteria [25]. The patients were admitted to the NICU of the First Affiliated Hospital of the University of Science and Technology of China between 2016 and 2022. Inclusion criteria: preterm infants born before 32 weeks of gestation and/or birthweight < 1500 g. Exclusion criteria included severe pneumonia, sepsis, pulmonary and cardiovascular developmental abnormalities, and those who received antibiotics and/or corticosteroids in their first 2 weeks, necrotizing enterocolitis (NEC), and Grade III/IV intracranial hemorrhage. Details of these patients at birth were displayed in Table S1.

### 2.2 BALF Collection From Clinical Samples

BALF samples were collected from preterm infants who were undergoing mechnical ventilation and were categorized into BPD (*n* = 28) and non‐BPD (*n* = 18) groups according to the 2001 NICHD diagnostic criteria for BPD. Details of these patients at birth were displayed in Table S2. The inclusion and exclusion criteria were the same as described in Section 2.1. BALF was collected as part of the conventional airway nursing procedure for clearing the respiratory tract, both at the time of intubation and at specific intervals (approximately 7 days after intubation) during mechanical ventilation. The procedure [26] involved administering 1 mL of sterile normal saline (NS) to the infants twice via endotracheal intubation, with a 30‐s pause between each administration. A neonatal suction catheter (6F, KG Corporation, Shanghai, China) was carefully inserted through the endotracheal tube, and approximately 0.7 mL of the lavage solution was aspirated back into a sterile sputum collector connected to the catheter. The lavage solutions were then transferred to sterile EP tubes and centrifuged at 450 g for 8 min at room temperature. The supernatant was stored at −80°C for further experiments. The sediment was passed through a 200‐mesh filter, and 20 μL was taken for absolute cell counting. The remaining cells were used for staining.

### 2.3 Flow Cytometry

Cells collected from the BALF of preterm infants were stained using the fluorochrome‐conjugated antibodies (Table 1) [27]. Briefly, staining was performed for 20 min in the dark, followed by washing with phosphate‐buffered saline (PBS) and centrifuging for 5 min at 450 g, 4°C. The cells were then acquired on a flow cytometer (NovoCyte Flow Cytometer 3130, Agilent Technologies Inc., USA). The data were analyzed using NovoExpress software. In terms of clinical blood samples, we use the residual samples after routine blood tests for detection. Due to the limited volume of remaining blood samples, only one of the following assays could be performed: platelet activity testing, plasma collection for cytokine analysis, or lymphocyte isolation for flow cytometry.

**Table 1:** Reagents and antibodies used in the study.

	Production	Cat.	Clone
FITC Mouse Anti‐Human CD66b	BD Pharmingen	5555724	G10F5
BV421 Mouse Anti‐Human CD62L	BD Pharmingen	563862	DREG‐56
Brilliant Violet 510 anti‐human CD45 Antibody	Biolegend	368526	2d1
FITC Mouse Anti‐Human CD41a	BD Pharmingen	555466	HIP8
PE Mouse Anti‐Human CD62P	BD Pharmingen	561921	AK‐4
APC‐Cy7 Mouse Anti‐Human CD14	BD Pharmingen	557831	(MφP9)
Mouse Monoclonal Integrin alpha 2b/CD41 Antibody ‐Alexa Fluor488	Novus	NB500‐549AF488	HIP8
FITC Mouse Anti‐Rat CD11b	BD Pharmingen	554982	(WT.5)
APC‐Cy7 Mouse Anti‐Rat CD45	BD Pharmingen	561586	OX‐1
PE‐Mouse Anti‐Rat CD62p	Santa Cruz	sc‐8419PE	(CTB201)
PAF ELISA Kit	Genie	UNFI0061	
Human IL‐6 Precoated ELISA Kit	DAKEWE	1110602	
Human IL‐8 Precoated ELISA Kit	DAKEWE	1110802	
Human GM‐CSF Precoated ELISA Kit	DAKEWE	1117302	
Human MPO‐DNA ELISA kit	MEIMIAN	MM‐2467H2	
Human GRO ELISA	RayBiotech	ELH‐GRO	
Human sL‐Selectin ELISA Kit	MUTI SCIENCES	EK185	
Rat IL‐6 ELISA Kit	MUTI SCIENCES	EK306	
Rat IL‐1αELISA Kit	MUTI SCIENCES	EK301A	
Rat IL‐18 ELISA Kit	MUTI SCIENCES	EK318	
Rat TNF‐αELISA Kit	MUTI SCIENCES	EK382	
Rat KC/CXCL1 ELISA Kit	MUTI SCIENCES	EK 396	
Rat IL‐1β ELISA Kit	MUTI SCIENCES	EK301B	
Rat Interleukin 12, IL‐12/P70 Elisa Kit	CUSABIO	CSB‐E07364r	
Rat Interleukin 33, IL‐33 Elisa Kit	CUSABIO	CSB‐E14077r	

Therefore, the number of samples tested for each indicator cannot be exactly the same.

### 2.4 Neutrophils Isolating and Stimulation With PAF

Human neutrophils were isolated from fresh peripheral blood of healthy neonates according to the instructions of the peripheral blood neutrophil isolation kit (Cat^#^P9040, Beijing Solarbio Science & Technology Co. Ltd). The isolated neutrophils were counted and placed in a 24‐well plate, stimulated with PAF (Cat^#^ HY‐108635, MCE) at 100 ng/mL for 0.5, 2, and 4 h. Then, some neutrophils were collected at each time point for flow cytometry detection. Some neutrophils were lysed in 1 mL of Trizol reagent and stored at −80°C until RNA extraction. Meanwhile, we divided the fresh neutrophils into three groups: neutrophils + PBS group, neutrophils + PAF group, and neutrophils + PMA (Cat^#^ HY‐18739, MCE) group. After stimulating with PAF or PMA and incubating for 4 h, we stained the neutrophils with MPO and Sytox green, and then observed and captured images under a fluorescence microscope.

### 2.5 Cytokines Analysis

The proinflammatory cytokine levels in BALF of preterm infants and lung tissue homogenate supernatants of rats were examined using ELISA (Table 1).

### 2.6 RNA Extraction and Reverse Transcription

Lung tissue RNA was extracted using TRIzol, and the concentration and purity of the obtained RNA were determined using the Nano OneDrop instrument. For reverse transcription, 100 ng of total RNA was used per reaction and converted to cDNA on ice using a two‐step method according to the instructions of MonScript RTIII All‐in‐One Mix with dsDNase (REF: MR05101, Monad Biotech Co. Ltd). Real‐time fluorescence quantitative PCR was performed using SYBR Green Premix Pro Taq HS qPCR Kit (Cat^#^ AG11701). Primers were listed in Table 2.

**Table 2:** Primers for qRT‐PCR.

Gene	Forward primer (5′−3′)	Reverse primer (5′−3′)	Amplicon size (bp)	Accession number
*IL6*	TTCGGTCCAGTTGCCTTCTC	CTGAGATGCCGTCGAGGATG	172	NM_000600.5
*LCN2*	TCAAGATCACCCTCTACGGGA	CGGCACCTGTGCACTCA	154	NM_005564.5
*IL10*	CCTGCCTAACATGCTTCGAGA	TCTTGGTTCTCAGCTTGGGG	198	NM_000572.3
*MMP12*	TCCTCACTGCTGTTCACGAG	GGATTTGGCAAGCGTTGGTT	184	NM_002426.6
*ACTB*	ACCTAACTTGCGCAGAAAACA	GTCCTCGGCCACATTGTGAA	175	NM_001101.5
*Il6*	TGATGGATGCTACCAAACTGGA	TGTGACTCCAGCTTATCTCTTGG	197	NM_001314054.1
*Il1ra*	CCTGAAATGGCAGTCGCTAGT	TCTGAAGGCTTGCATCTTGC	167	NM_001039701.3
*Il1b*	GCCACCTTTTGACAGTGATGAG	AGCTTCTCCACAGCCACAAT	186	NM_008361.4
*Plaur*	GCTTTCCACCGAATGGCTTC	TAACTCCGGTTTCCCAGCAC	151	NM_011113.4
*Gapdh*	CCCTTAAGAGGGATGCTGCC	ATGAAGGGGTCGTTGATGGC	197	NM_001289726.2

### 2.7 Neonatal Hyperoxia‐Induced Lung Injury Rat Model Establishment

Twelve healthy adult pregnant SPF Sprague Dawley (SD) rats, weighing 250–300 g, were individually housed in cages. The room temperature and humidity were maintained at 22 ± 2°C and 55 ± 10%, respectively. Each pregnant rat typically gave birth to 8–10 pups. Then these pups were randomly assigned to three experimental groups: Group 1 (hyperoxia group), Group 2 (hyperoxia with GB treatment group), and Group 3 (normoxic air group), with six pups in each group. The randomization process was completed through a computer‐generated random number sequence, ensuring that each animal had an equal probability of being assigned to any group. Dams were rotated between room air and hyperoxia every 24 h to avoid oxygen toxicity. The newborn rats were exposed to 85% O_2_ immediately after birth to mimic BPD, which is the classical method to establish a BPD animal model [28].

The animal experiments were conducted in triplicate. Rat body weights were recorded daily. Rats in Groups 1 and 2 were exposed to 85%–90% O_2_, while those in Group 3 were maintained in a normoxic environment (21% O_2_). The oxygen flow rate in the oxygen chamber was set at 1–3 L/min. The oxygen concentration within the chamber was continuously monitored over a 24‐h period using a medical digital oxygen monitor. Starting on Day 4 of the experiment, rats in Group 2 received intraperitoneal injections of GB at a dose of 10 mg/kg for 10 consecutive days. On Day 14, the rats were sacrificed, and their lung tissues were harvested and fixed in 4% formaldehyde. All animal procedures were conducted in accordance with the Declaration of Helsinki guidelines for the use and care of laboratory animals.

### 2.8 BALF and Blood Collection From Rats

BALF was collected as previously described. Briefly, 75% alcohol infiltrates the neck fur and cut open layers of neck skin to expose the trachea, followed by an indwelling needle connected to a 5 mL syringe to extract 1 mL of 0.9% saline and puncture it into the trachea, and then ligated with a silk thread. The ice‐cold PBS (0.5 mL) was instilled twice into the right lungs, and then at least 0.7 mL BALF was pulled back to the syringe. Then, the collected BALF was centrifuged at 3500 r/min, 4°C for 5 min to pellet the cells, and the supernatant was kept at −80°C until it was used for cytokine analysis. We collected peripheral blood from rats using orbital blood sampling. Newborn rats weighed only ~25 g at 14 days of age, so the collectible blood volume is very limited.

### 2.9 Lung Tissue HE Staining

The left lungs of BPD model rats were fixed overnight in 4% paraformaldehyde at 4°C and processed by successive dehydration with an alcohol gradient and xylene. The tissues were then embedded in paraffin and cut into 4‐µm‐thick sections for H&E staining or IHC. H&E staining was carried out as previously described to determine the alveolar numbers per field. Three rats were selected from each group, and three pathological sections were prepared from each lung tissue. Three fields of view were taken from each section, and the number of alveoli was counted. The tissue sections were analyzed by two experienced blinded pathologists.

### 2.10 The Homogenate Supernatant of Lung Tissue

Lung tissue (100 mg) was added with 1 mL buffer and three grinding beads, homogenized at 60 Hz for 2 min. The homogenate was acquired after centrifuging at 750 g for 10 min and stored at −80°C for later use.

### 2.11 RNA Sequencing

Total RNA was extracted using TRIzol reagent (Invitrogen, CA, USA) according to the manufacturer's instructions. RNA purity and concentration were evaluated using the NanoDrop 2000 spectrophotometer (Thermo Scientific, USA). The transcriptome sequencing and analysis were performed by OE Biotech Co. Ltd. (Shanghai, China).

### 2.12 Western Blotting for Lung Tissue

Lung tissues were lysed in Cell lysis buffer (Cat^#^ P0013, Beyotime, China) at a ratio of 10 mg:100 μL and 50× protease inhibitor and 50× phosphatase inhibitor, mixed with 3 mm grinding beads, 60 Hz × grind twice for 60 s, on ice for 25 min, and then centrifuged at 4°C, 16,000×*g* for 5 min, and the supernatant was taken 10 μL for protein quantification according to protein concentration determination according to the instructions of the enhanced BCA Protein Assay Kit (Cat^#^P0010S, Beyotime, China). Samples were then separated by 10% Bis‐Tris PAGE electrophoresis and transferred to a PVDF membrane for detection. Western blots were probed overnight at 4°C with specific primary antibodies (1:1000) in Tris‐Buffered Saline Tween‐20 (TBST) containing 5% skim milk. After washing three times for 10 min with TBST, the membranes were incubated for 1 h at room temperature with a respective IgG‐HRP‐labeled second antibody (1:3000) in TBST containing 5% skim milk. Antigens were revealed using a SuperSignal^TM^ West Pico PLUS Chemiluminescent (Thermo Scientific, USA). Antibodies were listed in Table 3.

**Table 3:** Antibodies used in Western Blotting.

Antibodies	Production	Cat^#^	Dilution
PhosphoPlus Jak2 (Tyr1007/Tyr1008) Antibody Duet	Cell Signaling Technology^TM^	8224S	1:1000
PhosphoPlus Stat3 (Tyr705) Antibody Duet	Cell Signaling Technology^TM^	8204	1:1000

### 2.13 Immunofluorescence

Paraffin‐embedded segments were used to evaluate the expression of IL‐6 in neutrophils (MPO) and macrophages (F4/80) according to protocols [29]. To obtain color images, a 40× laser scanning confocal microscope (Olympus FV1200, Tokyo, Japan) was utilized. Three rats were selected from each group. Three lung tissue sections were prepared from each rat for immunofluorescence staining. For each section, two fields of view were selected separately from the upper, lower, left, right, and central areas (40× magnification), and the number of positive cells in each field was counted. The average number of positive cells per area in the merge section was calculated and compared among different groups.

### 2.14 Statistical Analysis

The data were expressed as the means ± standard deviation (SD) and assessed for statistical significance using Student's *t*‐test when the data are assumed Gaussian distribution. Mann–Whitney test was used for the data without normal distribution. The one‐way ANOVA was used for comparisons between multiple groups. Values with a two‐tailed *p*‐value < 0.05 were considered statistically significant. The statistical analyses were performed using Graphpad Prism 9.3 software. As the animal experiments were conducted in triplicate, six pups per group, the data from three experiments were pooled for statistical analysis.

### 2.15 Study Approval

The clinical study was approved by the ethics committee of the First Affiliated Hospital of USTC (2021KY‐080‐A). Written informed consent was received, and informed consent has been retained. The animal experiment was approved by the experimental animal ethics committee of the First Affiliated Hospital of the University of Science and Technology of China (2022‐N(A)−082).

## 3 Results

### 3.1 Neutrophils and Platelet Activation in Infants With BPD

**Figure 1:**
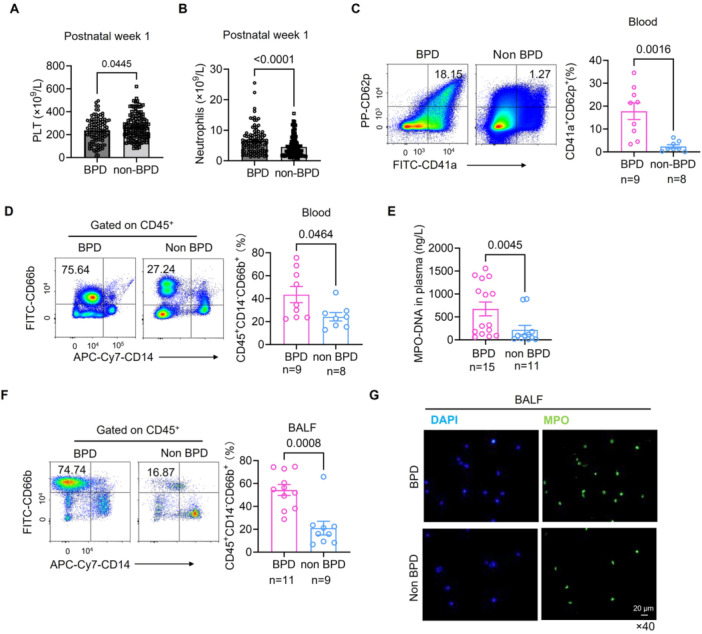
Neutrophils and platelets were activated in preterm infants with BPD. (A) Counts of platelet were lower in preterm infants with BPD (*n* = 100) compared with non‐BPD patients (*n* = 126) at postnatal Week 1. (B) Counts of neutrophils were higher in preterm infants with BPD (*n* = 100) compared with non‐BPD patients (*n* = 126) at postnatal Week 1. (C) CD62p increased higher in platelets in preterm infants with BPD (*n* = 9) than that in non‐BPD patients (*n* = 8). (D) The percentage of CD14^−^CD66b^+^ increased sharply in BPD patients (*n* = 9) than that in non‐BPD patients (*n* = 8). (E) The level of MPO‐DNA was elevated higher in plasma in BPD (*n* = 15) than in non‐BPD (*n* = 11). (F) In BALF from patients with BPD (*n* = 11), the percentage of CD45^+^CD14^−^CD66b^+^ was significantly higher than that in non‐BPD group (*n* = 9). (G) MPO was observed more in BALF of patients with BPD than in that of non‐BPD by immunofluorescence (magnification: 40×).

We discovered that platelets exhibited heightened activation, as evidenced by the increased expression of CD62p in the BPD group, despite a lower platelet count at postnatal Week 1. Concurrently, the absolute number of neutrophils was significantly higher and activated, as indicated by the marked increase in the proportion of CD66b at postnatal Week 1 in infants with BPD compared to those without BPD (Figure 1A–D). Additionally, we observed elevated levels of MPO‐DNA in the plasma of infants with BPD (Figure 1E), suggesting that activated neutrophils may be forming neutrophil extracellular traps (NETs), which could contribute to the pathogenesis of BPD [30, 31, 32]. Further investigation of the local alveolar microenvironment revealed a significantly higher proportion of CD14^−^CD66b^+^ cells in the BALF of BPD patients (Figure 1F), indicating that a large number of activated neutrophils had accumulated in the airways. Moreover, MPO expression was higher in the BALF of infants with BPD than in non‐BPD infants (Figure 1G).

**Figure 2:**
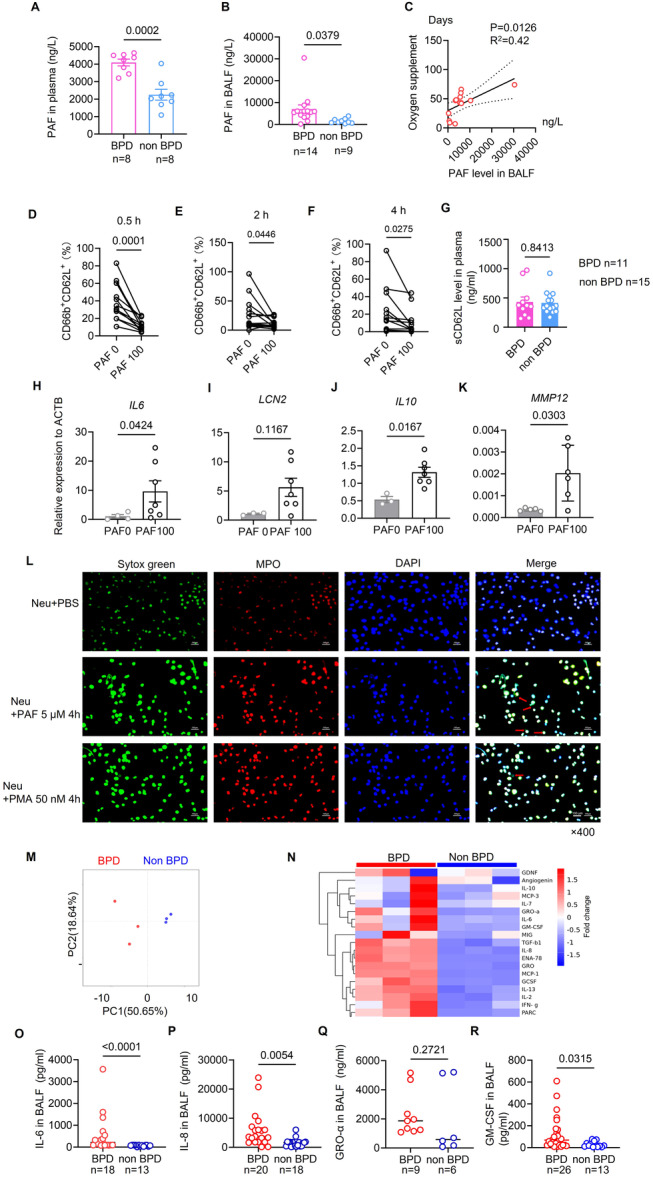
PAF stimulates neutrophils to be resident locally and secrete IL‐6. (A) The level of PAF in plasma from preterm neonates with BPD (*n* = 8) was higher than that in non‐BPD group (*n* = 8). (B) The level of PAF in BALF from preterm neonates with BPD (*n* = 14) increased higher than that in non‐BPD group (*n* = 9). (C) PAF level in BALF was positively correlated with oxygen supplement days in patients with BPD (*n* = 14). (D–F) The percentages of CD66b^+^CD62L^+^ in neutrophils from the blood of healthy neonates at birth decreased sharply after PAF (100 ng/mL) stimulation at different time points. (G) The level of soluble CD62L in the plasma of patients with BPD (*n* = 11) or without BPD (*n* = 15). (H–K) Relative mRNA expressions of *IL6*, *LCN2*, *IL10*, and *MMP12* were examined in neutrophils after PAF (100 ng/mL) stimulation for 4 h. (L) PAF induced neutrophils to form NETs (MPO represents neutrophil activation, and the red arrows indicate the presence of free DNA outside the cells, which signifies the formation of NETs). (M) Clustering of inflammatory cytokine levels in BALF from preterm neonates with and without BPD. (N) Differential inflammatory cytokines in BALF from patients with and without BPD were displayed in heatmap. (O–R) The levels of IL‐6, IL‐8, and GM‐CSF were tested higher in BALF of BPD patients (*n* = 9–26) postnatal 1 week than those of non‐BPD (*n* = 6–18), except for GRO‐α by ELISA. Each experiment was independently repeated at least three times.

To identify potential triggers for platelet activation, we measured PAF levels in plasma and found that PAF was significantly elevated in BPD infants compared to non‐BPD infants, with a similar trend observed in BALF (Figure 2A,B). Notably, PAF levels in BALF were positively correlated with the duration of oxygen supplementation, suggesting a close relationship between PAF and airway inflammation (Figure 2C).

To further elucidate the relationship between PAF and neutrophils, we isolated neutrophils from the fresh peripheral blood of healthy newborn infants and stimulated them with 100 ng/mL PAF at various time points. Our observations revealed that the proportion of CD66b^+^CD62L^+^ neutrophils began to decrease as early as 0.5 h after PAF stimulation and continued to decline significantly at 2 and 4 h post‐stimulation (Figure 2D–F). CD62L shedding is a hallmark of neutrophil diapedesis into peripheral tissues [33, 34], so it is inferred that high concentrations of PAF can promote the local retention of neutrophils in lung tissue, thereby limiting their migration into the bloodstream. However, there was no significant difference in sCD62L level between the two groups (Figure 2G). The levels of sCD62L may be influenced by gestational age, absolute neutrophil count (ANC) in peripheral venous blood within 6 h after birth, multiple pregnancies, and mode of delivery [35]. Therefore, the levels of sCD62L are not solely determined by the shedding of neutrophils. We then investigated the expression of several inflammatory cytokine genes in neutrophils and found that the relative mRNA levels of *IL6, IL10*, and *MMP12* were significantly upregulated following PAF stimulation, while *LCN2* exhibited an increasing trend (Figure 2H–K). Interestingly, we found after 4 h of stimulation with PAF, neutrophils are induced to produce NETs (Figure 2L), which was considered to promote BPD development [32].

Furthermore, protein array analysis revealed that four of the top ten upregulated proteins in the BALF supernatant (IL‐6, GM‐CSF, IL‐8, and GRO) were predominantly inflammatory cytokines associated with neutrophils (Figure 2M,N). We subsequently confirmed that IL‐6, IL‐8, along with GM‐CSF, were elevated in the BALF of infants with BPD compared to those without BPD (Figure 2O–R).

### 3.2 GB Treatment Relieved Lung Damage in the Hyperoxia Rat Model

**Figure 3:**
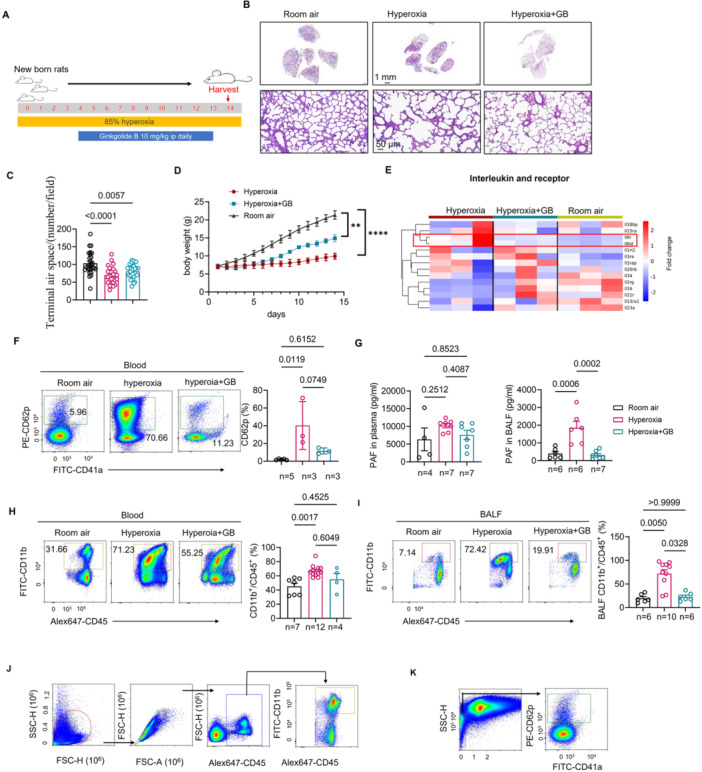
GB reduced hyperoxia lung injury in neonatal rats. (A) Protocol of animal model establishment. (B) HE staining of rat lung tissue was shown. (C) Terminal air space was observed and counted. (D) Weight change of the rats in three groups. (E) Rat lung tissue from Hyperoxia, Hyperoxia+GB and room air group was subjected to RNA sequencing analysis (three rats per group), and the differentially expressed genes of interleukins were displayed using a heatmap. (F) The comparison of percentages of CD41a^+^CD62p^+^ from the blood of the rats in the room air group (*n* = 5), hyperoxia group (*n* = 3), and hyperoxia with GB treatment group (*n* = 3). (G) Comparisons of PAF level in plasma and BALF of rats in three groups. Room air group (*n* = 4–6), hyperoxia group (*n* = 6–7), hyperoxia with GB treatment group (*n* = 7). (H) Comparison of CD45^+^CD11b^+^ frequency in blood from rats in different groups. Room air group (*n* = 7), hyperoxia group (*n* = 12), and hyperoxia with GB treatment group (*n* = 4). (I) Comparison of CD45^+^CD11b^+^ frequency in BALF from rats in different groups. Room air group (*n* = 6), hyperoxia group (*n* = 10), and hyperoxia with GB treatment group (*n* = 6). (J) Gate strategy of flow cytometry of CD45^+^CD11b^+^ frequency in blood and BALF from rats. (K) Gate strategy of flow cytometry of CD41a^+^CD62p^+^ in blood from rats. Six rats per group, and data from each group were repeated three times in each experiment.

Next, we aimed to determine whether the PAF receptor antagonist GB could alleviate alveolar inflammation and, if so, how it exerted this effect. To further confirm that blocking PAF could mitigate inflammation initiated by neutrophils, we established a neonatal rat model of hyperoxia‐induced lung injury mimicking BPD (Figure 3A). Histological examination of lung tissue via H&E staining revealed an enlarged alveolar volume in the hyperoxia group, a finding that terminal air space per field was reversed to some extent by GB administration (Figure 3B,C). The weight of the hyperoxia‐exposed rats progressively decreased over time; however, this weight loss was attenuated following GB intervention (Figure 3D). In the RNA sequencing analysis of lung tissues from three groups (Room air, Hyperoxia, and Hyperoxia+GB) of rats, the results regarding the differential expression of interleukin family genes indicated that the expression of *Il6st* and *Il6r* was both downregulated in the GB intervention group. However, no significant downregulation was observed for interleukin‐1‐related receptors (*Il1r2l* and *Il1rap*) (Figure 3E). This suggests that GB may exert its anti‐inflammatory effects by influencing the downstream signaling pathways of IL‐6 rather than IL‐1. The proportion of CD62p in the blood of hyperoxia‐exposed rats significantly increased but showed a decreasing trend after GB intervention (Figure 3F). Additionally, compared to rats maintained in room air, those in the hyperoxia group exhibited increased levels of PAF and a higher proportion of CD11b in both plasma and BALF. As anticipated, GB intervention led to a marked decline in PAF levels and the proportion of CD11b^+^/CD45^+^ cells in BALF (Figure 3G–I), as determined by the gating strategy used in flow cytometry analysis (Figure 3J,K), but there was no reduction compared to the normal group. This means that GB will reduce the number of inflammatory neutrophils, but will not reduce the number of normal phenotype neutrophils. It can be interpreted as GB block PAF, leading to limiting the rolling and adhesion of neutrophils, thereby reducing the migration of neutrophils from the peripheral blood to the local inflammatory site [36]. Collectively, these results suggest that GB exerts a beneficial effect on hyperoxia‐induced lung injury in neonatal rats.

**Figure 4:**
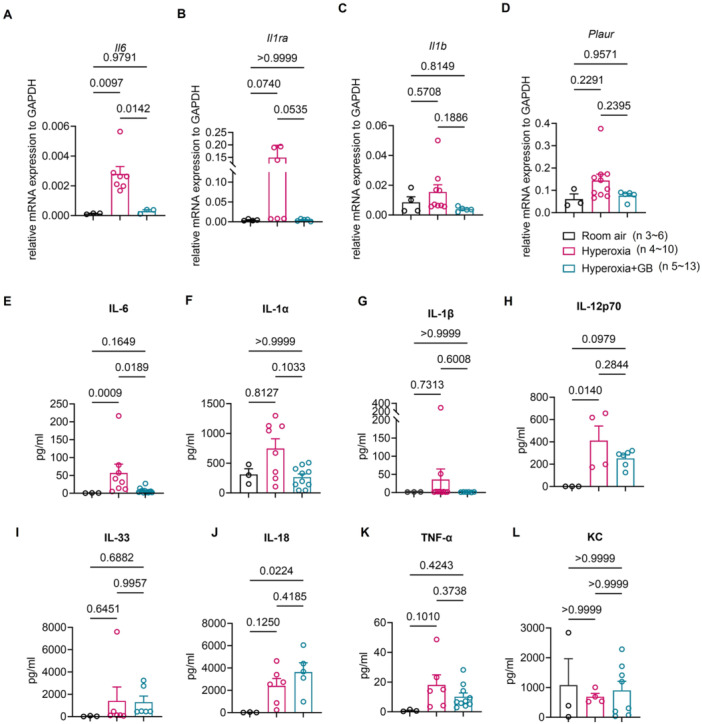
Relative mRNA expression of inflammatory genes and levels of cytokines in the lung tissue of rats in each group. (A–D) Relative mRNA expressions of *Il6, Il1ra, Il1b*, and *Plaur* of lung tissue in the different groups of rats. (E–L) The levels of IL‐6, IL‐1α, IL‐1β, IL‐12p70, IL‐33, IL‐18, TNF‐α, and KC in lung tissue homogenate of rats in three groups were examined. Six rats per group, and data from each group were repeated three times in each experiment. The data were pooled together for analysis. Room air group (*n* = 3–6), hyperoxia group (*n* = 4–10), hyperoxia with GB treatment group (*n* = 5–13).

Next, we sought to identify the inflammatory cytokines downregulated by GB intervention and observed significant downregulation of *Il6* gene expression along with the descending trend of *Il1ra*. While *Il1b* and *Plaur* also exhibited a downward trend, these changes did not reach statistical significance (Figure 4A–D). Furthermore, in the supernatant of lung homogenates, we also detected a marked decrease in IL‐6 levels. Additionally, levels of IL‐1α, IL‐1β, IL‐12p70, IL‐33, IL‐18, TNF‐α, and KC were reduced to varying degrees but without significant differences (Figure 4E–L). Collectively, these findings indicate that GB intervention can effectively downregulate the pulmonary inflammatory response in newborn rats with hyperoxia‐induced lung injury. During the experimental period, Group 1 (hyperoxia group) experienced the loss of four animals, with two deaths occurring on Day 7, the third on Day 9, and the fourth on Day 10. In Group 2 (hyperoxia with GB treatment group), three rats died on Days 7, 9, and 12, respectively. There was no death in Group 3 (normoxic air group).

### 3.3 GB Treatment Reduced Neutrophils‐Producing IL‐6

**Figure 5:**
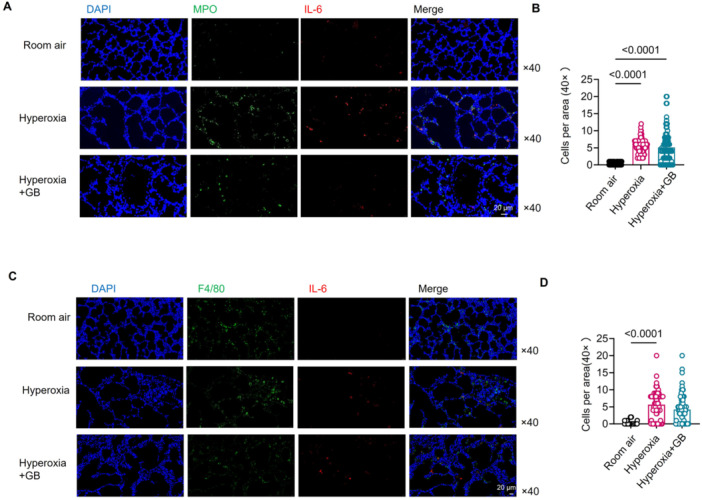
GB can reduce neutrophil‐producing IL‐6 in the hyperoxia lung tissue of rats. (A) Detection of MPO and IL‐6 co‐expression in lung tissues of rats in each group using immunofluorescence (MPO stands for activated neutrophils in rats) (magnification: 40×). (B) The comparison of the number of positive cells (MPO^+^IL‐6^+^) per field of view in merged images from lung tissues of three rats per group. (C) Detection of F4/80 and IL‐6 co‐expression in lung tissues of rats in each group using immunofluorescence (magnification: 40×). (D) The comparison of the number of positive cells (F4/80^+^IL‐6^+^) per field of view in merged images from lung tissues of three rats per group.

To confirm that IL‐6 is primarily produced by activated neutrophils in the hyperoxia lung injury model, we performed multiple immunofluorescence stainings for neutrophils and IL‐6 expression in lung tissue across different groups. We observed a significant co‐localization of MPO (activated neutrophil marker) and IL‐6 in the lung tissue of rats exposed to hyperoxia. However, after GB intervention, the expression of IL‐6 in neutrophils was markedly reduced (Figure 5A,B). In contrast, there was also some co‐expression of F4/80 (a macrophage marker) and IL‐6 cells in the lung tissue of hyperoxia‐exposed rats, but after GB intervention, the co‐localization of F4/80 and IL‐6 was not changed (Figure 5C,D). These results suggest that GB alleviates hyperoxia‐induced lung injury by inhibiting PAF‐stimulated neutrophils from producing IL‐6 rather than inhibiting macrophage‐derived IL‐6 locally. IL‐6 is a key factor involved in inflammation, autoimmunity, and cancer, and its effects are primarily mediated through the IL‐6‐signal transducer (Il6st) and the signal transducer and activator of transcription 3 (STAT3) pathway [37, 38].

### 3.4 GB Treatment Reduced IL‐6 Downstream Signaling Pathway

**Figure 6:**
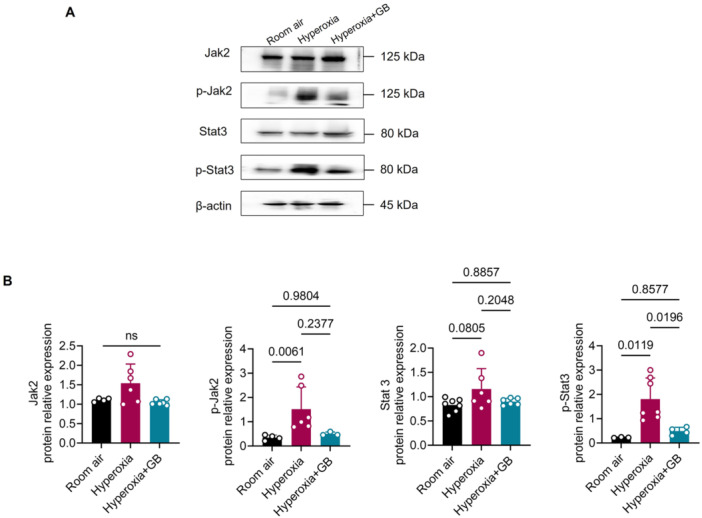
Western blot detection of Jak2, Stat3, and phosphorylation. (A) Western blot images of Jak2, Stat3, p‐Jak2, and p‐Stat3 in rat lung tissue in three groups. (B) Protein relative expressions of Jak2, Stat3, p‐Jak2, and p‐Stat3 in rat lung tissue in three groups using Image J software. Six rats per group, repeated three times in each experiment, and the data were pooled. Room air (*n* = 3–7), hyperoxia (*n* = 6–7), hyperoxia +GB (*n* = 3–6).

To further investigate whether GB intervention affects the IL‐6 signaling pathway, we conducted Western blot analysis. We found that the levels of phosphorylated Jak2 (pJak2) and phosphorylated Stat3 (pStat3) were significantly elevated in the hyperoxia group but were downregulated in the GB treatment group (Figure 6A,B). These findings further confirm that GB intervention can effectively downregulate the IL6‐Stat3 signaling pathway in hyperoxia‐induced lung injury.

## 4 Discussion

BPD is a multifactorial chronic lung disease and a significant respiratory complication in premature infants, profoundly impacting their long‐term lung function. Given its severe implications, elucidating its pathogenesis and developing novel treatment strategies are of utmost urgency. In this study, we discovered that neutrophils activated by PAF play a crucial role in the development of BPD. GB, a PAF receptor antagonist, can mitigate hyperoxia‐induced lung injury in neonatal rats. Specifically, GB alleviated the inflammatory response and downregulated the IL‐6/Stat3 signaling in neonatal rats with hyperoxia‐induced lung injury.

In our investigation, we observed higher PAF concentrations in the peripheral blood and alveolar compartments of infants with BPD, which is reported to correlate with disease severity [39]. Meanwhile, our ex vivo experiments revealed that PAF promotes neutrophil retention in the alveolar microenvironment by decreasing the expression of CD62L on neutrophils. Lower CD62L levels can signify neutrophil priming and heighten sensitivity to activation [40], potentially enhancing the extravasation of systemic neutrophils into the alveolar compartment and to release inflammatory cytokines locally, as evidenced by upregulation of the mRNA expressions of *IL6*, *IL10*, and *MMP12* genes involved in BPD. These findings, coupled with activation of neutrophils in BALF from BPD infants in our clinical samples—linked to inflammation, oxygen supplementation duration, and toxicity [37, 41, 42, 43]—suggested that PAF may be a pivotal inflammatory cytokine mediating immune cell activation in the alveolar microenvironment in BPD pathogenesis. PAF is a potent inflammatory mediator with widespread biological activity, synthesized and secreted by various cells involved in inflammatory reactions [44, 45]. It has been shown to trigger neutrophil depolarization [46, 47] and induce changes in neutrophil size, membrane potential, and intracellular pH, inhibiting degranulation in sepsis models [48]. PAF‐producing neutrophils are reported to be key players in disease progression during anaphylaxis [49]. Consistent with these findings, our work identifies the PAF–neutrophil co‐function as the pivotal driver of inflammation in BPD, thereby offering a novel therapeutic clue for this disease. Further research is needed to elucidate the detailed mechanisms and intracellular signaling pathways involved in PAF‐mediated neutrophil activation.

Notably, we detected elevated IL‐6 levels both in the BALF of preterm infants with BPD and in lung homogenate supernatant in rats of hyperoxia groups, which indicates that IL‐6 plays a critical pathogenic role in the onset and progression of BPD, similar to the report in umbilical cord blood [44, 50, 51]. IL‐6 can promote the proliferation, migration, and inflammatory responses of alveolar epithelial cells and pulmonary arterial smooth muscle cells (PASMCs) by activating the JAK–STAT3 signaling pathway via the gp130 protein, thereby impairing alveolarization and angiogenesis, leading to pulmonary vascular remodeling and pulmonary hypertension (PH) contributing to BPD [52]. IL‐6 signaling, activated by hyperoxia, is associated with the loss of alveolar epithelial Type II cells (ATII), and inhibition of IL‐6 signaling could promote alveolarization and ATII cell survival after hyperoxia [50, 53].

We also observed that, in the hyperoxia group, the co‐expression of MPO and IL‐6 in lung tissue was greater than that of F4/80 (macrophage markers) with IL‐6, indicating that pulmonary IL‐6 could be predominantly derived from neutrophils in this animal model, which was in accordance with the fact that neutrophils have been identified as a principal source of IL‐6 that fuels trans‐signaling and aggravates pulmonary inflammation [54, 55]. Following GB intervention, pulmonary co‐expression of MPO with IL‐6 was markedly reduced in lung tissue, whereas IL‐6 levels in F4/80‐positive cells remained unchanged. These findings indicated that neutrophil‐derived IL‐6 could be the principal driver of pulmonary inflammation in hyperoxia‐induced lung injury, and that GB exerted its protective effect primarily by suppressing IL‐6 production in neutrophils rather than in macrophages. Furthermore, *Il6r* and *Il6st* mRNA gene expressions in the lung tissue of GB‐treated rats were also downregulated, along with less expression of Jak, Stat3, and their phosphorylation levels, elucidating that GB could suppress neutrophil‐IL‐6‐Stat3 and alleviate hyperoxia lung injury. Likewise, in an OVA‐induced BALB/c asthma model, GB markedly downregulated both transcription and secretion of IL‐6 from bronchial epithelial cells [56]. In cultured human pulmonary microvascular endothelial cells (HPMECs), GB can block IL‐6–induced up‐regulation of vascular cell adhesion molecule‐1 (VCAM‐1) and intercellular adhesion molecule‐1 (ICAM‐1), cutting neutrophil adhesion to weaken inflammation [57].

However, GB did not appear to inhibit the expression of *Il1ra*, *Il1b*, and *Plaur* mRNA in lung tissue, contrasting with its effects in the nervous system [58]. This discrepancy may be explained by the different origins of inflammatory cytokines from various immune cells in different organs, as neutrophils rarely infiltrate the nervous system under sterile conditions. Besides, in a neonatal rat model of hypoxic‐ischemic brain injury (mimicking perinatal hypoxic stress), intraperitoneal (i.p.) administration of GB (1–10 mg/kg) for 14 consecutive days did not increase mortality, and significantly ameliorated organ injury without detectable acute toxicity [59, 60].

Totally, our findings suggest that PAF stimulates neutrophil activation and IL‐6 secretion, a critical link in the inflammatory response to hyperoxia‐induced lung injury. This process can be partially blocked by GB administration, highlighting GB's potent anti‐inflammatory effects in hyperoxia‐induced lung injury.

Thus, we inferred that GB confers protection in a spectrum of pulmonary disorders probably via curbing IL‐6 production at the source, interrupting IL‐6 signal transduction, and dampening the ensuing IL‐6‐driven inflammatory cascade. This study has several limitations. It remains unclear how GB influences other local immune cell populations in the lung, and whether it contributes to the repair of injured pulmonary stromal cells; long‐term impacts on alveolar development and lung function were not assessed. Future studies are needed to address these gaps and further clarify GB's therapeutic potential in neonatal lung injury.

## 5 Conclusion

PAF receptor antagonists can effectively mitigate hyperoxia‐induced lung injury in rats and inhibit the production of IL‐6 by neutrophils, along with downregulating the IL‐6/STAT3 inflammatory signaling pathway in lung tissue. These findings suggest that targeting the PAF receptor may hold promise as a therapeutic strategy for the treatment of BPD. Future research should focus on clinical trials to evaluate the safety and efficacy of GB in preterm infants at risk of BPD, as well as exploring the detailed mechanisms underlying its anti‐inflammatory effects.

## Author Contributions

Conceptualization: Xuesong Wang and ShuShu Wang. Data curation: Mingwu Chen. Formal analysis: Yong Lv and Yue Qi. Funding acquisition: Xuesong Wang. Methodology: Xuesong Wang and Dake Huang. Supervision: ShuShu Wang. Validation: Xuesong Wang and ShuShu Wang. Writing – original draft: Xuesong Wang. Writing – review and editing: Xuesong Wang and ShuShu Wang.

## Consent

Informed consent was obtained in writing from a parent or legal guardian for every preterm infant enrolled.

## Conflicts of Interest

The authors declare no conflicts of interest.

## Supporting information


**Table S1:** Clinical characteristics of preterm neonates with or without BPD. **Table S2:** Clinical characteristics of preterm neonates with or without BPD.
