# Supplementary material for: Ginkgolide B Alleviates Airway Inflammation in Hyperoxia Lung Injury
Source: Immun Inflamm Dis. 2026 Apr 2;14(4):e70364. doi: 10.1002/iid3.70364 (PMC13045252; doi:10.1002/iid3.70364)
Supplement: Supplementary file 1 — Table S1: Clinical characteristics of preterm neonates with or without BPD. Table S2: Clinical characteristics of preterm neonates with or without BPD. [file IID3-14-e70364-s001.docx]

**Table S1** Clinical characteristics of preterm neonates with or without BPD

|  | BPD(n=100) | Non-BPD(n=126) | P value |
| --- | --- | --- | --- |
| Gestational Age (wks) | 28.60±1.27 | 31.60±2.06 | <0.001 |
| Birth weight(g) | 1111±166.9 | 1301±173.7 | <0.001 |
| Male (n) | 56 | 49 | 0.0034 |
| Asphyxia(n) | 82 | 86 | 0.0216 |
| Mechanical ventilation(n) | 35 | 5 | <0.001 |
| Non-invasive ventilation(n) | 54 | 21 | <0.001 |

**Table S2** Clinical characteristics of preterm neonates with or without BPD

|  | BPD (n= 28) | Non-BPD (n=18) | P value |
| --- | --- | --- | --- |
| Gestational Age (wks) | 29.66±1.30 | 30.28±0.93 | 0.0474 |
| Birth weight(g) | 1118±184.4 | 1234±181.0 | 0.0402 |
| Male (n) | 15 | 6 | 0.2318 |
| Asphyxia (n) | 22 | 11 | 0.3146 |
| Mechanical ventilation (n) | 10 | 1 | 0.0899 |
| Non-invasive ventilation (n) | 8 | 8 | 0.3470 |
